# Precision dentistry- Way line to periodontics

**DOI:** 10.6026/973206300212293

**Published:** 2025-08-31

**Authors:** Aarudra Devi, Nikita Ravi, Anitha Logaranjani, Jaideep Mahendra

**Affiliations:** 1 Department of periodontics, Meenakshi Ammal Dental College and Hospital, Chennai-600095, Tamil Nadu, India

**Keywords:** Precision dentistry, predictive modelling, risk assessment, genome sequencing, diagnostic medicine

## Abstract

Dentistry is stuck between the one-size-fits-all approach towards diagnostics and therapy employed for a century and the era of stratified medicine. The present review presents the concept of precision dentistry, *i.e.*, the next step beyond stratification into risk groups and lays out where we stand, but also what challenges we have ahead for precision dentistry to come true. Current approaches for enabling more precise diagnostics and therapies focus on stratification of individuals using clinical or social risk factors or indicators. Precision dentistry refers to tailoring diagnostics and therapy to an individual; it builds on modelling, prediction making and rigorous testing. Most studies in the dental domain focus on showing associations and do not attempt to make any predictions.

## Background:

In recent decades, the development of high-throughput technologies has made it possible to develop rapid, reliable and less expensive methods for complete DNA sequencing, so much so that it has led to a real revolution in genetic research, increasing its large-scale use [1]. Whole Genome Sequencing (WGS), or Whole Exome Sequencing (WES), *i.e.*, sequencing of a coding nucleotide portion of the genome, qualitative and quantitative analysis of messenger RNA (transcriptome), analysis of the epigenetic influences of the genome that vary its expression (epigenome) and analysis of metabolites (metabolome) and proteins (proteome) in relation also to the eubiotic or dysbiotic environment (micro biome) are termed "omics" sciences [2]. Precision dentistry is a multifaceted, data driven approach to oral and dental health care that uses individual genetic data to segregate and stratify individuals with similar genetic makeup into phenotypic groups to deliver precise or targeted treatment. The aforementioned approach to disease will eliminate the undue side effects of the conventional treatment which targets the 'average patient' [3]. The stark understanding of precision dentistry will necessitate sufficient global data collection, initial introducing technology for data collection, bio-banking, framing of data safety protocols, designing easy data access to corresponding individuals and measures to implement genetic data to clinical practice. Once achieved, this will necessitate the introduction of the concept of 'precision dentistry' to the dental education system [4]. Therefore, it is of interest to report precision dentistry too will advance, taking cues from medical practices in their pursuit of precision medicine. Precision dentistry is the future and it is closer than you believe.

## Genomic sequencing:

Genomic sequencing is a Tran's formative technology and effective integration in healthcare requires system-wide change. 2 Beyond the technical requirements of establishing sequencing and bioinformatics capacity to process samples, the real barriers to widespread clinical implementation span diverse domains, including data integration and interpretation, workforce capacity and capability, public acceptability and government engagement, paucity of evidence for clinical utility and cost effective ness and ethical and legislative issues [3, 4]. Frameworks for implementing genomic-medicine programs in single institutions and multi-institution collaborative are available, [2, 5] but information on translating this experience to transform whole healthcare systems is scarce. The ultimate goal of translational genomics research is to use knowledge gained from genomics research to improve the precision of the practice of medicine at individual level and to inform public health strategies at population level. Thus, proper implementation of genomic-driven insights into the pathobiology of diseases and therapeutics promises to contribute to better health in all human populations. Advances in genomics research are enabling the implementation of next-generation sequencing (NGS) in 21st century western medical care, particularly in areas of tumor screening, family health history-directed decision support, pharmacogenomics-driven treatment, complex disease risk advice and diagnostic genome sequencing [1]. In contrast, genome-based decision support for public health is absent or inadequate in most low and middle income countries (LMIC). Of 603 international laboratories offering genetic testing registered in Gene Tests, none are in low income countries and only 20 are found in middle-income countries (www.genetests.org). The hurdles encountered by LMIC in the move towards genomic medicine include inadequately equipped research and clinical laboratories, shortage of scientifically skilled personnel, lack of awareness of the importance of genomics to guide public health and lagging public health policy framework. Recent initiatives such as the Human Heredity and Health in Africa (H3Africa), the Qatar Genome Project and the Mexico National Institute of Genomic Medicine (INMEGEN) are fostering genomic research capacity of LMIC by funding locally-initiated research and empowering local researchers to lead genomics research projects [5]. Successful examples include the African Genome Variation Documenting the striking landscape of genetic structure of diverse ethnic groups with the goal of facilitating genomic medicine in Africa and Mexico [6].

## Elements of precision medicine:

Low-cost precision medicine driven by genomic research and technological advances present tremendous promise for LMIC because the morbidity and mortality cost of common diseases is disproportionally high in resource limited settings. Therefore, early investment in genomic research has the potential for returning long-term benefits to LMIC. Sequencing and genotyping costs are decreasing by at least ten fold per year (www.genome.gov/images/ content/cost_per_megabase2.jpg) thanks to technological advances. In the near future, NGS based screening programs are anticipated to be more cost-effective than conventional clinical tests currently in use in LMIC. This is good news to LMIC in terms of affordable genomics research and clinical practice. Moreover, LMIC can maximize the impact of available resources by pooling funds to establish and strengthen regional research centers instead of working in isolation. More generally, the disproportionate paucity of pharmacogenomics research in LMIC has limited our knowledge of clinically relevant variants in non-European ancestry populations [14]. This picture is beginning to change with the growing pool of pharmacogenomics research [15, 16-17] and supportive programs such as the Pharmacogenomics for Every Nation Initiative (PGENI) that aims to integrate pharmacogenetics with the public health system of LMIC by constructing a resource for relevant polymorphisms using DNA samples from major ethnic groups that represent at least 10% of the population in LMIC. The emergence of PM associated with the complexity of "omics" sciences "catalyzes new organizational concepts in the Health Services system, compels investment in highly specialized personnel capable of using high-throughput technologies and producing, reading and assembling big data derived from genomics [6]. The emergence of PM associated with the complexity of "omics" sciences "catalyzes new organizational concepts in the Health Services system, compels investment in highly specialized personnel capable of using high-throughput technologies and producing, reading and assembling big data derived from genomics [6].

## Current progressive action of precision medicine:

PM is currently also being applied to oral cavity diseases and therefore referred to as "precision dentistry" (PD). PM is currently also being applied to oral cavity diseases and therefore referred to as "precision dentistry" (PD). Particularly, PD is finding great use in the study of oral cavity tumors, which account for 50% of head-neck cancers. Particularly, DP is finding great use in the study of oral cavity tumors, which account for 50% of head-neck cancers [7, 8- 9]. D Phase produced valuable findings regarding squamous cell carcinoma (SCC). SCC accounts for 90% of oral carcinomas. Treatment is multidisciplinary: surgery, radiotherapy and chemotherapy are often then accompanied by adverse outcomes, with patient death within five years of onset [10]. Although the early stage of SCC predisposes invasion") [11, 12, 13, 14-15]. DP has produced valuable findings regarding squamous cell carcinoma (SCC). SCC accounts for 90% of oral carcinomas. Treatment is multidisciplinary: surgery, radiotherapy and chemotherapy are often then accompanied by adverse outcomes, with patient death within five years of onset [10]. Although the early stage of SCC predisposes better than the advanced stage, there is still a mortality rate of 13% to 30%.

## Prediction models used in dentistry:

As laid out, prediction modelling is at the heart of precision dentistry. A range of prediction models have been developed: predicting (1) caries increment (number of new lesions or progressing ones on patient level, termed caries risk), (2) periodontal disease onset or progression (incidence of periodontitis, or worsening periodontal lesions or extent or stage on patient level, termed periodontitis risk); (3) progression of a specific caries lesion (termed caries lesion activity); (4) tooth loss (mainly in periodontitis patients and mainly during supportive periodontal care). All models are supposed to assist the practitioner in making more precise treatment planning decisions. Sir, in 2015, US President Barack Obama announced the launch of the Precision Medicine initiative. Given that genetics, environment, lifestyle and diet all have an impact on health; it is an excellent endeavour that takes into account the distinctive biological blueprint that each human is born with. The National Research Council's Toward Precision Medicine adopted the definition of precision medicine as 'The tailoring of medical treatment to the individual characteristics of each patient to classify individuals into sub-populations that differ in their susceptibility to a particular disease or their response to a specific treatment. Preventative or therapeutic interventions can then be concentrated on those who will benefit, sparing expense and side effects for those who will not (3).

## Caries risk assessment:

Knowledge about future caries increment would, in daily care, allow assigning specific interventions (*e.g.*, tailoring a supportive oral program and deducing individualized intervals for supportive re-evaluations, targeting possible risk behaviors or traits). A wide range of models have been developed, building on both risk factors causally associated with disease incidence and progression, like diet, oral hygiene, or fluoride intake; and risk indicators like past caries experience (a surrogate for behavioral patterns, genetic [17].

## Periodontitis risk assessment:

Like caries, having knowledge about an individual's future periodontitis onset or progression, could help to tailor active periodontal care as well as the intensity and interval of supportive care. A systematic review from 2015 summarized the available tools [9]. Nineteen studies were included; six of them showed low risk of bias. A total of five risk assessment tools were identified. The most often investigated tool was the Periodontal Risk Assessment and its modifications, assessed by twelve studies. Six publications dealt with the DenPlan Excel/Previsor Patient Assessment and its modifications; the remaining tools were assessed by only 1-3 studies [12]. Again, the different instruments employed a similar set of possible risk indicators or factors, while the number and weighting of them differed. The review stated that the instruments were able to discriminate individuals with different probability of disease progression, while one needs to highlight that overall; the validation of the instruments was limited, marred by high risk of bias and inconsistency. The review did not allow for synthesis and robust conclusions [17].

## Reason why do we fail:

Why we may fail so why do prediction models in dentistry show only some usefulness (caries risk), unclear usefulness (periodontitis risk), or no usefulness at all (tooth loss) - after decades of research and more and more powerful software and hardware. To make predictions? Why are we obviously quite some distance away from precision dentistry? While it is not fully clear, one potential source of this problem lies with the risk factors and indicators we build these models on: They are either clinically determined and capture the phenotype of the unit of interest (a patient, a tooth, a surface, a pocket) or are recorded from patient history and questionnaires (on his or her diet, oral hygiene behavior etc.). In a nutshell, one could argue that oral and dental research has built models on those parameters that we as experts think are important. Moreover, we often employ parameters captured in a single time point (usually the base line visit) and do not reflect changes in the status of risk indicators or factors over time. In a recent study on claims data, we tried to overcome both aspects to some degree: In that study, we predicted mortality using predictor variables from a dataset of over 40,000 potential risk factors and indicators, many of them repeatedly collected from over 300,000 individuals (unpublished). Prediction was possible with high (and useful) accuracy. Notably, the most important risk indicators were not diseases or any specific treatments provided, but unexpected ones like the costs for transporting individuals or the fact that individuals consumed inpatient instead of outpatient care. As outlined below, using such large datasets, with longitudinally collected and broad data may allow overcoming the current constraints in prediction modelling. The technologies to harness these data (compute, algorithms, storage) are available; the main question.

## Complexities and shortcomings of conventional diagnostic approaches:

The advancements in biomedical technology, particularly regarding high-throughput methods, sensitive diagnostic platforms and machine learning algorithms, have undermined the limitations of the conventional diagnostic approaches [7]. The advent of the keystone pathogen hypothesis has made understanding of the periodontal microbiome better [8]. It has been observed that the keystone pathogen Pophyromonas gingivalis has the propensity to alter the milieu of a periodontal pocket from having symbiotic microflora to a dysbiotic one [9]. The emphasis is now focused on the interaction between the dysbiotic microbiome and inflammatory mediators that cause periodontitis [11]. The risk factors for periodontitis include environmental, genetic and systemic factors. Viral and fungal infections also deplete the immune response of the host and individuals with an over reactive genetic predisposition are predisposed to severe forms of periodontitis. As the disease is caused by a multitude of factors, traditional diagnostic measures fall short of assessing the disease comprehensively. The advent of biomarkers has made the diagnosis of periodontitis much easier and more specific.

## Advantages of precision over traditional periodontal diagnosis and treatment:

A precision periodontal diagnosis enables the customization of disease-prevention strategies based on an individual's unique genetic, environmental and lifestyle factors. It facilitates the prescription of more effective drugs that are tailored to an individual's specific genetic makeup. Precision medicine reduces the time, cost and failure rate of pharmaceutical clinical trials by identifying responders and non-responders to a particular therapy. Finally, it shifts the emphasis in medicine from reaction to prevention by predicting susceptibility to disease and improving disease detection. It eliminates trial-and-error inefficiencies that inflate healthcare costs and undermine patient care [16].

## Biomarkers in precision periodontics:

Predictive Markers These help clinicians pinpoint patients at risk of disease, enabling proactive modification of screening protocols and risk factor adjustments to maximize disease prevention efforts. Single nucleotide polymorphisms (SNPs) are the best example of predictive markers, as they can identify if the patient is genetically susceptible to disease [15]. A study conducted by Schulz *et al.* [18] on SNPs in the IL-1 gene cluster and sub gingival colonization of microorganisms observed that the genetic makeup of the IL-1 gene cluster correlated with the likelihood of Aggregatibacter actinomycetemcomitans colonization in sub gingival areas. It was concluded that there is no evidence to state that this correlation is an independent risk indicator for periodontitis [17].

## Prognostic markers:

These are assessed upon disease onset and do not require temporal variation. Commonly utilized markers are genetic. These biomarkers serve as a valuable tool in treatment planning, enabling clinicians to predict potential issues, select the most effective treatment pathway and establish a tailored maintenance plan to ensure long-term stability and optimal patient outcomes [18]. Prognostic markers are measured when disease occurs; they do not need to change over time and they serve to estimate disease characteristics and the stage and grade, which are indispensable for accurate prognostics of the progression pattern and responsiveness to different treatment protocols [19]. A meta-analysis conducted by Beck et al (5) revealed that the IL-1A (-889C/T) polymorphism was significantly associated with an increased susceptibility to chronic periodontitis in African, European and American populations [20].

## Diagnostic markers:

This group encompasses biochemical and microbiological markers, as they can identify various important parameters related to the disease activity. To assess the patient's compliance with treatment, surrogate markers, including inflammatory, tissue response products and bone health markers, are indicated and categorized in this group [22].

## Soft tissue biomarkers:

These markers serve as tissue degradation indicators, which include matrix metallo proteinases (MMPs). Both MMP8 and MMP9 levels are increased in periodontitis. Also, both MMP13 and MMP8 have been observed to be elevated in patients with peri implantitis. Platelet-derived growth factor (PDGF) supports healing and increased vascular endothelial growth factor (VEGF) expression in epithelial cells and endothelial cells in periodontitis-affected gingiva may be a useful marker for periodontal healing [19]. A meta-analysis by Ghassib *et al.* [11] examined the use of biomarkers in peri-implant crevicular fluid (PICF) to distinguish between healthy implants, peri-implant mucositis and peri-implantitis. They found that pro inflammatory cytokines such as IL-1β and IL-6 in PICF can be used as adjunct tools to clinical parameters to accurately identify healthy implants from those with peri-implant mucositis or peri-implantitis.

## Main challenges:

A number of challenges for implementing and sustaining data-driven applications have been identified and apply to dentistry, too.

## Concerns and confidence:

The balance between the public interest in attaining data and individual data protection demands has been handled differently across the globe. Questions around broad consent, data donation and cyber security but also around bias, fairness and responsibility of AI and other data-driven applications have been raised. Confidence in abstract and complex data products that support the dental workforce (*e.g.*, diagnostic support tools) and allow non-dental professionals to perform certain tasks (*e.g.*, dental screening via handheld devices in nursing homes by nurses) may grow over time as the public health benefits will become apparent. However, transparency, trustworthiness and explain ability are fundamental for the uptake of data-driven applications [21].

## Pitfalls, bias and failures:

For data-driven application in health care, there is reasonable concern about handing over critical medical decisions to computers. The stakes are high, as any treatment decision will affect patients' well-being. Failures of data-driven applications are most often rooted in biases that are not always apparent and therefore difficult to compensate. Sample selection bias (*i.e.*, training on non-representative, small and siloed data sets) leads to limitations in generalization, for example, on different devices or patient populations (Krois *et al.* 2021) [14]. Further bias originates from distributional shift in the target population; in such cases, data-driven systems may confidently make erroneous predictions based on "out-of-sample" inputs (Challen *et al.* 2020) [12]. Humans, including clinicians, are not perfect either and tend to give significance to evidence that supports their presumptions (confirmation bias). Automation bias and automation complacency, respectively, refer to phenomena where clinicians are in favor of accepting the guidance of an automated system, especially when challenged by multiple concurrent tasks (Parasuraman and Manzey 2010) [20].

## Capabilities:

Stakeholders' (*e.g.*, users' and consumers') capabilities toward adapting, employing and appraising data applications are currently limited. Educating the future health care workforce in data literacy seems crucial. Professionals need to be enabled to access, interpret, appraise, manage and ethically handle data (Calzada Prado and Marzal 2013); there is a call for the "data-driven physician" (Stanford Medicine 2020). In a survey of 523 US physicians and 210 medical students and residents, nearly three-quarters of medical students and nearly half of all physicians are planning to pursue additional education around data (*e.g.*, advanced statistics or data science), providing evidence that young professionals are aware of the future challenges for their profession and that training in this field is currently insufficient (Stanford Medicine 2020). Medical training and education need to keep pace with technological and data-driven developments. Notably, a good basic science background is already a prerequisite for entering dental and medical school, but little emphasis is placed on data analysis and applied mathematics. Data literacy should be a core competence in dental under- and postgraduate curricula. Introducing such new technologies into dental education is possible. One example for this is Computer-Assisted Design and Computer-Assisted Manufacturing (CAD-CAM): by now, many dentists routinely employ this technology and leverage their strengths while knowing how to cope with their weaknesses. Postgraduate courses on CAD-CAM are widely available and allow graduated dentists to learn and take up this technology, too. Data-driven applications are available on the market and have proven their additional value, postgraduate courses and training offers will become available and will educate an "informed user"-someone who is not necessarily an expert but can actively navigate the field. Furthermore, democratizing data science via automation will allow medical professionals and researchers to perform or replicate data science exercises on their own (*e.g.*, on open data), increase trust and bridge the gap between the technical and medical domains [22]. The data era and data dentistry will be a chance and not only a threat: it may help to push the profession toward a more critical and literate stance toward scientific data, as indicated by the exploding wealth of educational activities and their adoption by medical and dental professionals.

## Standardization:

Data exchange and usage require harmonization and standardization. Efforts toward systematizing medical terminology (*e.g.*, SNOMED: The Systematized Nomenclature of Medicine Clinical Terms, containing 300,000 uniquely identified, logically defined and hierarchically arranged terms, or MedDRA: Medical Dictionary for Regulatory Activities, a standardized medical terminology facilitating medical product regulatory information exchange) support the semantic interoperability of data, allowing cross-sectional data exchange. Public agencies, trusted institutions, or community-based initiatives may take a prominent role in establishing standards and benchmarking frameworks to ensure quality, generalizability and transparency of data-driven applications (International Telecommunication Union 2018.
